# Aesthetic outcomes of and anatomic reconstruction for Wassel type IV-D radial polydactyly using a modified Bilhaut-Cloquet procedure

**DOI:** 10.3389/fped.2023.1192168

**Published:** 2023-07-07

**Authors:** Zijing Du, Yan Cui, Hao Jiang, Dong Han

**Affiliations:** ^1^ Department of Plastic and Reconstructive Surgery, Shanghai Ninth People’s Hospital, Shanghai Jiao Tong University School of Medicine, Shanghai, China; ^2^ Department of Hand Surgery, Jinshan Hospital of Fudan University, Shanghai, China

**Keywords:** thumb duplication, Bilhaut-Cloquet, Wassel, type IV-D polydactyly, surgery

## Abstract

Wassel type IV-D thumb duplication is the most complex form and anatomic reconstruction is difficult. The aim of this study was to create an aesthetically satisfactory anatomical reconstruction for Wassel type IV-D radial polydactyly thumbs using the modified Bilhaut-Cloquet procedure. Surgery was performed on 24 thumbs with Wassel type IV-D radial polydactyly. To align the joint surfaces, the proximal phalanxes were unequally joined, primarily on the ulnar, and the distal phalanx was either symmetrically joined or unequally joined via curvature osteotomy. The patients were followed up for 12–91 months. The interphalangeal joint remained stable in all cases. The average functional score was 13.5 points (maximum 14 points). The overall average cosmetic score was 3.3 (maximum 4 points). Our modification of the Bihaut-Cloquet procedure produced good functional results for patients with Wassel type IV-D radial polydactyly. This method is used to correct the alignment and to stabilize the interphalangeal joint in both hypoplastic thumbs.

## Introduction

Congenital thumb duplication is the most common hand abnormality (1). Wassel first defined radial polydactyly and then described its seven types (2). Wassel Type IV is the most common type and is defined as thumb polydactyly that is duplicated at the metacarpophalangeal joint level. Hung et al. (3) proposed the following four subtypes of Wassel Type IV: (A) hypoplastic (12%), (B) ulnar deviated (64%), (C) divergent (15%), and (D) convergent (complex) (9%). Type IV-D is characterized by the most complicated bone and soft tissue anomalies. Its clinical manifestation is a zigzag deformity of the two thumbs, with divergence at the MCP and convergence at the IP joint. Both the radial and ulnar thumbs are symmetrical and hypoplastic. Reconstruction of thumbs with Type IV-D is significantly challenging due to the complexity of the deformity.

Regardless of which duplicated thumb is removed during treatment, the remaining thumb often exhibits a zigzag deformity. Various techniques are currently used to treat zigzag deformities, including open/closed wedge osteotomy of the metacarpal bone and/or proximal phalanx, flexor tendon centralization/relocation, oblique osteotomy, soft tissue reconstruction, and the Bilhaut-Cloquet procedure (4–15).

The Bilhaut-Cloquet (B-C) procedure is typically used when both thumbs are nearly equal in length and size and neither is thought to be sufficient for reconstruction on its own. In thumb duplication, the B-C procedure has well-known drawbacks, including a wider thumb, ridged nails, and a stiff joint. Although this method has some disadvantages, it remains critical to achieve a stable conclusion. To achieve good aesthetic and anatomic outcomes, modern reconstructive strategies incorporating components from both thumbs are now recommended.

In this study, a modified Bilhaut Cloquet procedure is used to reconstruct type IV-D thumbs with unequal combinations of multiple bones. The aim is to present the surgical techniques, report the outcomes, and evaluate the functional and aesthetic effects of this approach.

## Methods

### Patient study

We studied 24 thumbs of 24 patients with zigzag polydactyly. The patients were treated between January 2015 and August 2022 (Table 1). The average age of the patients was 17.1 months (range, 11–46 months), and the average follow-up time was 40.8 months (range, 12–91 months). Among the selected patients, 4 of them did not have their nails combined. This study protocol was approved by the institutional review board and the ethics committee of our hospital.

**Table 1:** Patient data.

Case	Gender	Affect site	Age at surgery (months)	Follow-up age (months)	Function points (14 points)	Cosmetic points (4 points)	Pain and satisfaction (2 points)	Total (20 points)	Postoperative nail width ratio (affected/contralateral)
1	F	R	10	36	13	3	2	18	1
2	M	R	14	57	14	4	2	20	1.04
3	F	L	13	43	14	4	2	20	1.09
4	F	L	46	12	14	3	2	19	1.09
5	F	R	11	51	14	3	2	19	1
6	M	L	18	42	14	4	2	19	1.2
7	M	L	16	41	14	3	2	19	0.75^a^
8	M	L	13	33	13	2	2	17	1.11
9	F	R	13	28	13	3	2	18	0.85^a^
10	M	R	12	91	14	4	2	20	1.12
11	M	L	11	27	13	3	2	18	1.07
12	F	L	14	84	13	3	2	18	1.1
13	M	R	12	60	14	3	2	19	1.14
14	M	R	31	46	13	3	2	18	1.08
15	F	R	24	32	13	4	2	19	0.78^a^
16	F	R	13	43	14	4	2	20	1.13
17	M	L	15	41	13	3	2	18	1.09
18	M	R	12	31	13	3	2	18	1.16
19	M	R	23	22	13	3	2	18	1.05
20	M	R	13	26	14	4	2	20	1.09
21	M	R	38	51	13	4	2	19	1.1
22	F	R	13	26	14	4	2	20	0.9^a^
23	M	R	13	15	14	4	2	20	1.09
24	M	L	13	41	13	3	2	18	1.03

^a^ The nail combined was not performed in case 7, 9, 15, 22.

### Surgical technique

**Figure 1:**
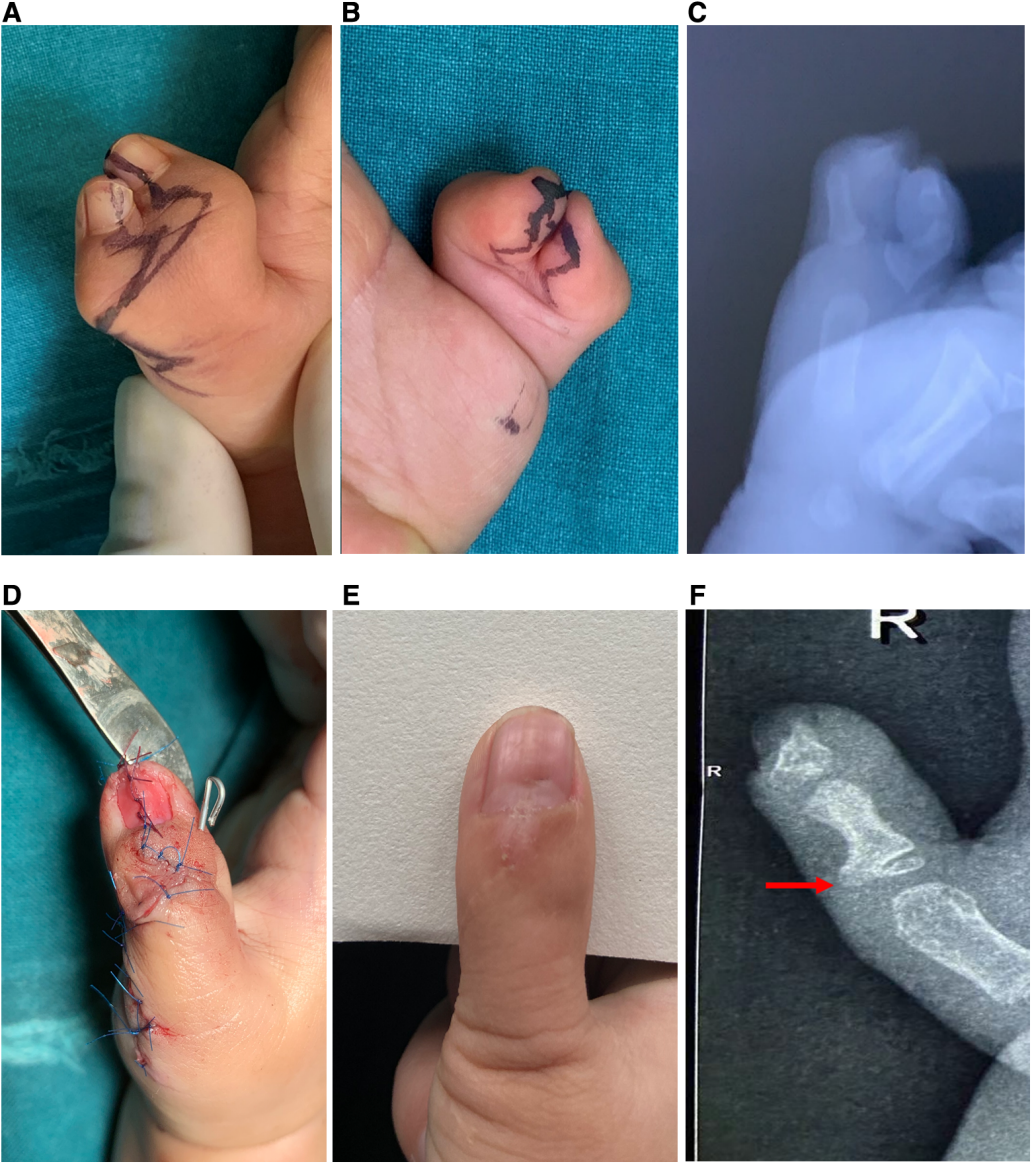
(**A,B**) Case 23, a lateral zig-zag incision; (**C**) preoperative x-ray; (**D**) postoperative appearance; (**E,F**) postoperative view, and postoperative x-ray after 15 months; red arrow shows radial protrusion of the proximal phalanx.

**Figure 2:**
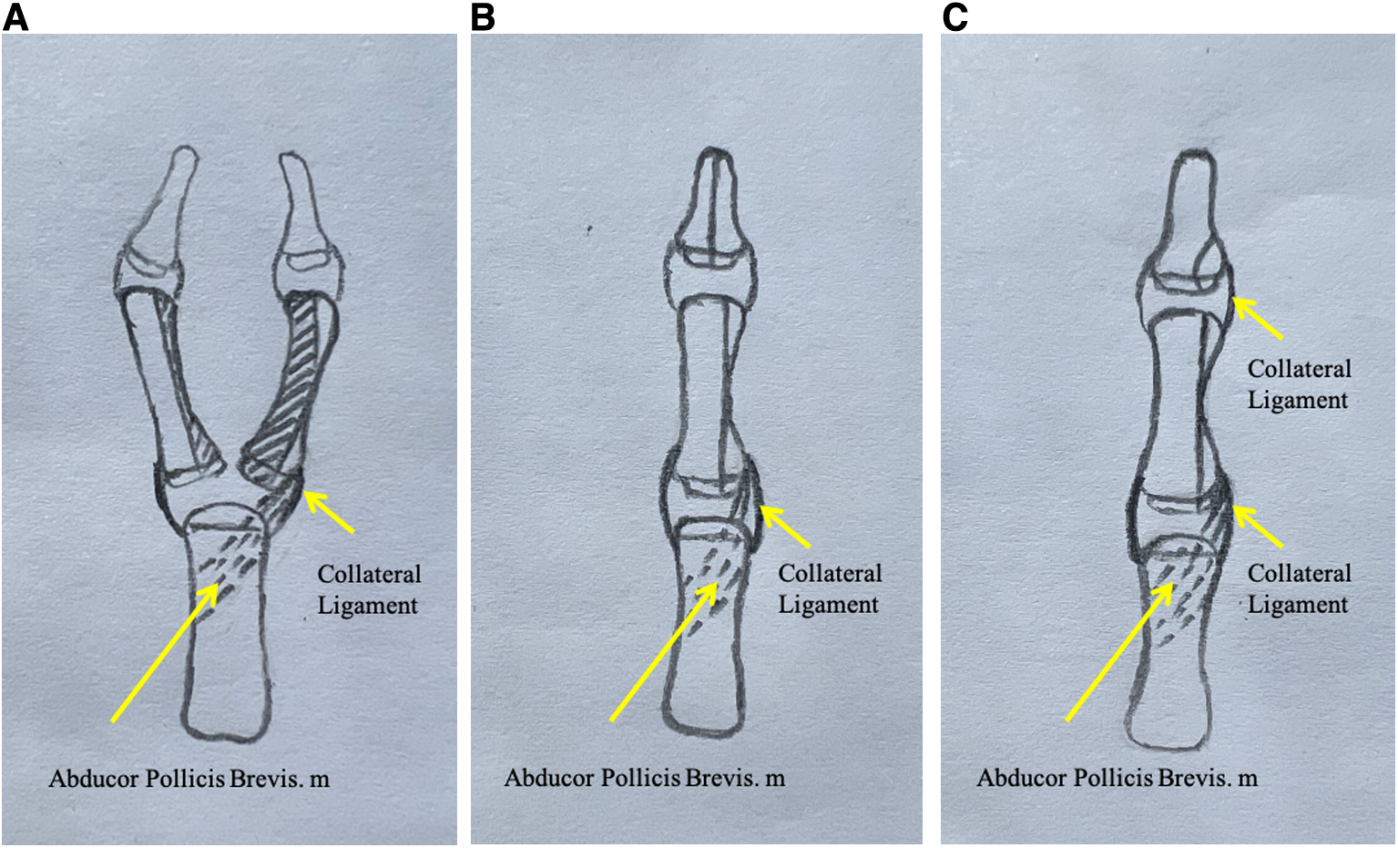
(**A**) Osteotomy of the proximal phalanx. The dark areas are resected, and the two proximal phalangeal bones are combined and their joint surfaces are aligned. (**B**) The halves of the distal phalanxes are joined. (**C**) Curved osteotomy of the distal phalanx is performed if the nails are not combined.

Before surgery, lateral zigzag incisions were drawn (Figures 1A,B). The reconstructed nail's width is measured in relation to the contralateral thumb. The ulnar nail remains intact if the nails are not combined. The central portion of the soft tissue and distal phalange are removed. The protruding part of the radial joint and the central cortex of the proximal ulnar phalanx are removed. Segmental osteotomy was performed on the radial proximal phalanx to prevent injuring the thenar muscle insertion. A Kirschner wire or 4-0 PDS sutures was utilized to fuse the preserved proximal phalange pieces (Figure 2). Align the joint surfaces on both sides of the proximal phalange and, if necessary, cut the reserved radial proximal phalange. A Kirschner wire or 5-0 PDS sutures was used to fuse the preserved distal phalanxes. To avoid causing a “seagull deformity”, a bone block was placed in the dip where the two distal phalanxes joined (16). If the ulnar nail was retained, the nail bed in the distal phalanx was peeled from radial to ulnar, the dark regions were excised, and the two distal phalanxes were fused using 4-0 PDS sutures or a Kirschner wire (Figure 2C). The detached FPL and EPL were fused, and the insertion was rebuilt to the middle of the distal phalanx base. The nail bed and matrix were stitched together with 6-0 absorbable sutures. The volar skin was covered with triangular skin flaps to avoid scar contracture and to create a smooth contour of the restored palm. The skin wound was closed with 6-0 PDS sutures. After surgery, the affected extremity was immobilized in an arm length cast for 6 weeks. Four to six weeks following surgery, the Kirschner wires were removed. Once the Kirschner wires were removed, the afflicted thumb was immobilized with a splint every night for 3 months. Young patients were required to participate in rehabilitative exercises for 3–6 months thereafter.

### Outcome evaluation

The Japanese Society for Hand Surgery Outcome was used to assess the outcome (Table 2). The joint stability was tested by applying lateral stress to the joint and then comparing its stress response to that of the contralateral thumb. A handheld goniometer was used to measure the motion range and joint deviation. Thumb alignments were assessed using anteroposterior x-rays of the thumb. Nail deformities and surgical scars were evaluated in terms of cosmetic appearance. The nail width of the reconstructed thumbs was measured and compared to that of the contralateral thumb.

**Table 2:** Scoring system for thumb duplication provided by the Japanese society for surgery of the hand.

Function	Points		Apperance		Points
Abnormal alignment			Size	Acceptable	1
IP joint	<5°	2		Unacceptable	0
	6°–20°	1	Finger pulp/nail	Acceptable	1
	>20°	0		Unacceptable	0
MCP joint	<10°	2	Surgical scar	Acceptable	1
	11°–19°	1		Unacceptable	0
	>20°	0	Bulging	None	1
Instabilty				Outstanding	0
IP joint	<10°	2	Subjective assessments		
	11°–19°	1	Pain	None	1
	>20°	0		Painful	0
MCP joint	<40°	2	Patients’ satisfaction	Satisfactory	1
	41°–59°	1		Unsatisfactory	0
	>60°	0	Total assessments		
Active flexion				Excellent	20
IP and MCP joint	>90°	2		Good	17–19
	60°–90°	1		Fair	14–16
	<60°	0		Poor	0–13
Extension lag					
IP and MCP joint	0°	2			
	<30°	1			
	>30°	0			
Palmar abduction					
MCP and CMC joint	<60°	2			
	31°–59°	1			
	<30°	0			

## Results

Table 1 shows the surgical outcomes of 24 patients. Early complications were not observed. The mean time to revision was 40.8 months (12–91 months). The average functional point score was 13.5 (maximum 14 points) on the Japanese Society for Surgery of the Hand evaluation form. There was no evidence of growth impairment or axis defects on radiographs. All the patients had satisfactory MCP joint alignment, stability, and range of motion. In general, the active range of motion of the MCP was similar to normal. There was no misalignment at the IP joint. In each case, the range of motion varied between 20° and 30° at the level of the IP joint. In case 9, an extension lag of less than 30° was detected in the IP joint. None of the patients’ parents reported that the patient experienced any discomfort or difficulty in their daily lives.

**Figure 3:**
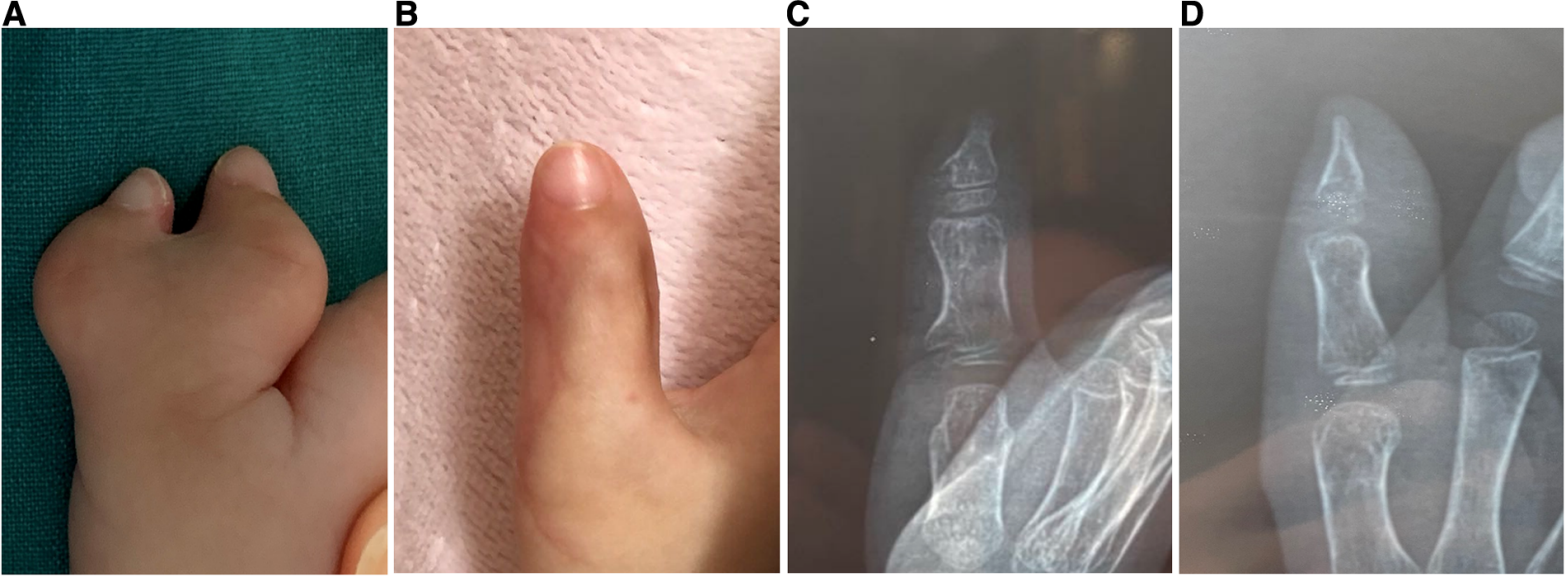
Case 9, (**A**) preoperative view. (**B–D**) Postoperative view and radiograph 28 months after none of the nails were combined.

The average cosmetic score was 3.3 points (maximum 4 points). Except for patients in which the nails were not combined, all the other patients showed nail union and an acceptable surgical scar. There was no evidence of a seagull deformity or nail splitting. The reconstructed nail was 0%–16% wider than the opposite side. After surgery, the patient's parents were satisfied with the appearance and function of the reconstructed thumb (Figures 1, 3).

## Discussion

More than 50% of polydactyly cases are Wassel Type IV polydactyly. Excision of the duplicate radial thumb produces good results in most cases of type IV duplications (1, 17). Despite the use of proximal phalanx and metacarpal bone osteotomy, type IV-D duplications, axis deviations, and joint instability still occur frequently (18, 19). IP joints are hypoplastic and unstable and frequently have rotational and fixed flexion deformities (3). The Bilhaut-Cloquet technique has been used to correct axis deviation and improve thumb stability, but the joint's range of motion is sacrificed, particularly in cases of nail dystrophy (17, 20–22). Tien et al. (10) described a soft tissue-only reconstruction method, including tendon centralization and A2 reconstruction. Abid et al. proposed an oblique osteotomy of the base of both distal phalanxes, which are combined by bone suture (5). And tendon relocation or tendon rebalancing was proposed for type IV-D duplications reconstruction (8, 9, 15).

In our technique, the proximal phalanx was resected with the central part and unequally assembled. The proximal phalanx of the ulnar side was used as the main trunk. The articular surfaces of the distal and proximal radial parts of the ulnar proximal phalanx, as well as the bone cortex of the radial side, were removed. Parts of the distal and proximal articular surfaces of the radial proximal phalanx were preserved. To align the articular surface, the retained radial articular is assembled on the ulnar side. The preserved proximal small radial bone block can be used to adjust the ulnar deviation of the proximal ulnar phalanx by including the insertion of the thenar eminence and avoiding metacarpal osteotomy of the metacarpophalangeal joints. Hung et al. used the method in which the radial proximal phalanx segment with the abductor pollicis brevis tendon insertion was transferred to the ulnar proximal phalanx (23). The radial segment, including the articular surface, bone, and insertion of the abductor pollicis brevis tendon into the radial side of the growth plate ulnar proximal phalanx, was preserved. The articular surface of the radial bone block is small, so there was little effect on metacarpophalangeal joint movement after being combined. All the patients had unrestricted movement of the metacarpophalangeal joints. He et al. found that a portion of the FPL was inserted into the lateral side of the distal phalanx (24). Interphalangeal joint dysplasia and tendon insertion abnormalities resulted in lateral deformities. We preserved the radial side with the articular surface, bone and joint capsule and the collateral ligaments distal to the ulnar proximal phalanx. During the follow-up, we found that on patient had a large residual radial bone mass. X-ray showed a radial protrusion (Figure 1F), which had a minimal effect on appearance. Excessive dissection of the radial part should be avoided during the operation because of potential bone resorption. Radial protrusion of the proximal phalanx may be related to continuous tension at the thenar muscle insertion.

If the nail needed to be combined, we combined both distal phalanxes. We used a small bone block to fill the phalanx depression to improve the curvature, as mentioned in the article describing a Type III duplication (16). For some patients whose nails were not combined or those with one nail that was 70% of the normal width, the preserved nail bed was completely peeled away from the distal phalanx bone surface, which was unequally combined, and then the nail bed was reset. Both methods can avoid causing nail deformities.

None of the patients experienced any significant limitation of thumb activity in daily life. The range of motion of the MCP is not significantly different from that of normal joints. The passive range of motion of the interphalangeal joint can reach 10°–40°, but the active range of motion is approximately 10°–20°. The interphalangeal joint moved well immediately after the operation. However, the range of motion gradually decreased over time. Postoperative immobilization may cause joint adhesion. Postoperative immobilization and early mobility are contradictory. The mismatch of artificial joint surfaces is also a factor that causes a decline in joint activity. Among them, one patient had limited extension, and extension lag may be related to the lengthening of the extensor tendon after the change in the force line.

The majority of parents were content with the appearance and functionality of the fingers. The reconstructed digit was bulky to various extents in some cases, owing to excessive swelling of the retained tissue. Swelling can be reduced by using elastic bandages.

## Conclusions

Patients who underwent the modified B-C procedure for type IV-D thumb duplication had a virtually normal reconstructed thumb and stable joint. The operation is more challenging and requires extensive experience as the operation plan often requires constant adjustment to obtain the best results. We hope that through continuous procedure optimization, we can help patients achieve good aesthetic and anatomic outcomes.

## Data Availability

The raw data supporting the conclusions of this article will be made available by the authors, without undue reservation.
